# Conference of Walter E Dandy Neurosurgical Society: My Life-Time Experience

**DOI:** 10.31729/jnma.4881

**Published:** 2020-03-31

**Authors:** Anita Poudel

**Affiliations:** 1 Nepal Army Institute of Health Sciences, Sanobharyang, Kathmandu, Nepal

## Abstract

I still remember

“Neurosurgery requires a commitment to one's own excellence and commitment to another's identity. The decision to operate at all involves an appraisal of one's own abilities, as well as a deep sense of who the patient is and what he/she holds dear”

- Dr. Paul Kalanithi

I have always wanted to be a doctor of some sort. During first year in dissection I was waiting for day when the skull would be dissected, as I found most interesting part of body were brain and heart. The human brain is most complex than any other known structure in universe. The complex network of glia and neurons give rise to every aspect of our shared humanity.^1^ Memory capacity of brain is reported to have equivalent of 2.5 petabytes of memory capacity.^2^

Neurosurgeons are in charge of the most amazing thing we know within all the universe-the human brain. In a medical life every medical student once has a dream of being a neurosurgeon. Although it gradually disappears with time. It is a complex specialty and it's methods, devices and procedures are being evolved and improved everyday. Thought of neurosurgery make my cardiac muscle pump blood through my vascular system really quickly. A dream I thought to quit like others but I couldn't quit it's calling on September 5, 2019. This day changed my thought about neurosurgery that it is possible. Only in our thoughts it is impossible. It was a conference conducted by Walter E Dandy Neurosurgical Society.^3^

Walking down through the memory lane, I still remember how excited me and my friends were for this first international conference. The preparation we did the day before was hilarious. It was a wonderful day of my third year that I got an opportunity to be a part of this conference. The conference was where I met excellent exemplars of neurosurgery. Dr. Saleem Abdulrauf who has helped develop high flow bypass surgery, a procedure for treating intracranial aneurysm was the host of conference.^4^ He beautifully presented about complex structure - the brain. This conference has challenged my thoughts about brain in many aspects. This conference was something that peeked every aspects of human brain function. It was higher functions like interpreting, vision, touch and as well as speech, emotions, learning, reasoning and fine control of movements. Even before we leave the womb, our brain works throughout our life to control our body's functions and help us to understand and interact with the world around us.^5^

I found out that neurosurgery appeals to those individuals who find human brain fascinating and who enjoy the physical act of correcting abnormalities of nervous system. Although the intellectual challenge of constant learning and change may draw an individual to neurosurgery, it must be coupled with a strong desire to be an interventionist, willing to make decisions and take responsibility for those decisions as here we are dealing with brain the boss of body. In every step we need to face a challenge. Much time is spent considering the various options before choosing an approach to a problem.

I also met Dr. Aneela Darbar6 only US trained female neurosurgeon from Pakistan. Talking to her made me feel as if I were on cloud nine. I also spoke to other female neurosurgeons. It was like a dream come true. What I loved about them was their dedication, hard work and patience. These empowered women were contributing to the health and prosperity of the patients and improved prospects for the upcoming generation. Education is one of the most important means of empowering women.

My experience in this conference can be summarized by the quote “A teacher takes a hand, opens a mind and touches a heart”. The dream of becoming a neurosurgeon, that can at times seen unobtainable, may be achieved with persistence, patience and hard work. Every individual's path to becoming a neurosurgeon is unique, but they all start with the same first step and positive thought in a mind.

This was what I learnt and experienced during the conference. It was a wonderful opportunity for aspiring students like us to gain a lot of information about field which we are scared or let's say in dilemma of choosing as a career.

## Conflict of Interest

**None.**
